# Enfoque local deliberativo de las controversias bioéticas: una oportunidad para la adecuada implementación de la tamización neonatal

**DOI:** 10.7705/biomedica.5313

**Published:** 2020-12-12

**Authors:** Ricardo A. Cifuentes

**Affiliations:** 1 Universidad Militar Nueva Granada, Bogotá, D.C., Colombia Universidad Militar Nueva Granada Universidad Militar Nueva Granada BogotáD.C Colombia

**Keywords:** tamización neonatal, bioética, autonomía personal, justicia social, beneficencia, obligaciones morales, Neonatal screening, bioethics, personal autonomy, social justice, beneficence, moral obligations

## Abstract

**Introducción.:**

Las características controversiales de la tamización neonatal influenciadas por consideraciones bioéticas hacen compleja su implementación. Colombia no es ajena a esta situación y las circunstancias locales complican el panorama.

**Objetivo.:**

Determinar cómo se abordan en el contexto local las controversias bioéticas en torno a la tamización neonatal como fundamento de las deliberaciones sobre el deber ser de esta actividad en Colombia.

Materiales y métodos.

Se aplicó una encuesta en el marco de un estudio interpretativo con dos componentes de análisis, uno descriptivo y otro deliberativo, en torno a los valores expuestos por funcionarios del Instituto Nacional de Salud.

**Resultados.:**

La oferta obligatoria de la tamización por parte de la nación, independientemente del costo de oportunidad y el consentimiento para el uso de sus resultados y de las muestras residuales en la investigación, no suscitaron controversias, pero sí el tipo de información y la autorización para hacer la tamización. Los funcionarios con mayor experiencia expresaron su preferencia por una tamización obligatoria (17,7 *Vs.* 11,79 años en promedio; p=0,007). Sorpresivamente, a pesar del riesgo de discriminación, teniendo como fin el neonato, hubo acuerdo en entregar toda la información a padres e historia clínica. Otro aspecto controversial fue la identificación de los pacientes en el seguimiento, frente a lo cual los funcionarios de mayor experiencia en aspectos bioéticos prefirieron el uso de códigos (4,5 Vs. 1,26 años en promedio; p=0,009). En este contexto, estrategias como el disentimiento informado, el asesoramiento especializado o los programas de salud pública que aprecien la diversidad permitirían rescatar valores, incluso aquellos aparentemente opuestos.

**Conclusión.:**

La aproximación local al deber ser de la tamización neonatal desde una perspectiva bioética deliberativa permitió ajustar una propuesta para su implementación.

## Abstract (en)

**Introduction:**

The controversial characteristics of neonatal screening influenced by bioethical considerations make its implementation complex. Colombia is not an exception in this sense and local circumstances complicate the panorama.

**Objective:**

To establish how bioethical controversies on neonatal screening are approached at a local level as a basis for deliberating on the must-be of this activity in Colombia.

**Materials and methods:**

A survey immersed in an interpretative investigation with descriptive and deliberative components of analysis was applied to approach the values exposed by officials of the Colombian *Instituto Nacional de Salud.*

**Results:**

The compulsory offer of screening by the nation, regardless of its opportunity cost and the consent for the use in research of results and residual samples, were not controversial, but, in contrast, the type of information and the consent to authorize screening did arise controversy. The more experienced officials preferred mandatory screening (17.7 vs. 11.79 years on average, p=0.007). Surprisingly, despite the risk of discrimination, keeping the neonate as the purpose, there was agreement on giving all the information to parents and medical records. Another controversial aspect was the follow-up of cases without hiding their identification where officials with more experience in bioethical aspects preferred the use of codes (4.5 vs. 1.26 years on average, p=0.009). In this context, strategies such as informed dissent, specialized advice or public health programs that appreciate diversity would allow to rescue even seemingly opposite values.

**Conclusion:**

A local approach regarding what ought to be in neonatal screening based on a deliberative bioethical perspective allowed to present an implementation proposal for this activity.

La tamización neonatal constituye un programa de salud pública que mediante la aplicación de pruebas, como las genéticas y moleculares en el momento del nacimiento, pretende detectar con prontitud anomalías que no son evidentes clínicamente. Su objetivo es el beneficio directo del recién nacido ^1^, aunque, gracias a los avances tecnológicos, en algunos casos puede ir en beneficio de terceros, como la familia o, incluso, la sociedad ^2^. En este contexto, las pruebas incluidas en los programas de tamización neonatal difieren según los países debido a consideraciones bioéticas que, en ocasiones, prevalecen frente a las científicas ^3^.

Algunas de las características de la tamización neonatal influenciadas por las consideraciones bioéticas a nivel internacional, incluyen: la búsqueda de equidad en el acceso ^4^ relacionada con el principio de justicia; la pretensión de ampliar los beneficios con la evaluación simultánea de decenas de anomalías a partir de una sola muestra de sangre ^5^; el asesoramiento de profesionales expertos ^6^; el seguimiento individual y de los programas ^7^ basadas en el principio de beneficencia; la mayor probabilidad de falsos positivos y daños asociados debido al elevado número de anomalías evaluadas ^5^, y la poca probabilidad de encontrar un sujeto con una anomalía metabólica frente al riesgo de estigmatizar a portadores exentos de mutaciones, lo que se relaciona con el principio de no maleficencia; la garantía de confidencialidad sobre los resultados ^8^ y de respeto a la privacidad de las muestras ^9^, y el derecho de los padres a conocer en qué consiste la prueba y tener la opción de rechazarla ^10^, lo cual se relaciona con el principio de autonomía.

Por otra parte, hay algunas características de la tamización neonatal también sujetas a consideraciones bioéticas que actualmente son objeto de debate. En cuanto a las pruebas de reconocido beneficio, se destacan los siguientes asuntos controversiales: si la oferta debe depender del costo de oportunidad ^11^; si la información de las pruebas de tamización debe ser general o detallada sobre cada anomalía ^7^^,^^12^; si hay necesidad de consentimiento informado para autorizar su realización ^10^; si deben revelarse los resultados de la tamización que no tengan un equilibrio riesgo-beneficio favorable para el neonato, como podría ser el caso de un portador sano ^13^; si el almacenamiento de muestras residuales puede ser a largo plazo ^9^, o si debe permitirse la utilización de las muestras y los resultados para la investigación. También, hay controversias con respecto a la realización de pruebas sin beneficio directo para el neonato, capaces de detectar anomalías poco comprendidas o sin posibilidad de intervención, a si deben realizarse por sugerencia de eminencias o en el contexto de investigaciones para obtener evidencia ^14^, o a si deben revelarse todos los resultados, aunque su significado sea incierto ^5^.

Colombia no es ajena a las controversias en torno a estos aspectos bioéticos, así como a circunstancias locales que hacen más complejo el panorama ^3^. En cuanto al aspecto legal, con la ley de tamización neonatal de 2019 se garantizó una tamización básica como mínimo ^15^, similar a la propuesta previa que consideraba un análisis de costos ^16^. Por otra parte, hay acuerdo en torno al derecho al diagnóstico y el tratamiento de todo niño con anomalías congénitas ^17^ mediante la tamización ampliada ^15^. En cuanto a la autonomía, la normatividad reconoce el derecho de los padres de los menores a consentir o rechazar procedimientos ^18^ y, específicamente en la tamización neonatal, a que quede registro escrito del consentimiento informado; además, el personal médico tiene la obligación de informar sobre la finalidad de dicha tamización y sus posibles consecuencias ^15^. La norma, sin embargo, no especifica la forma de proceder para el consentimiento, es decir, si la información debe ser general o específica sobre cada anomalía, y tampoco se indica cuáles resultados deben revelarse o cómo manejar las muestras residuales ^3^. Por otro lado, la información sobre la identidad de las personas puede ser utilizada por las autoridades sanitarias para la vigilancia ^19^, lo que implica el riesgo de generar estigma y discriminación de los afectados e, incluso, de los portadores sanos.

En este contexto, considerando la influencia de los principios éticos en la implementación de la tamización neonatal y las circunstancias locales relacionadas con ellos, el objetivo del presente estudio fue determinar cómo se abordan en el contexto local colombiano las controversias alimentadas por las consideraciones bioéticas, con el in de hacer una propuesta de base para la discusión en torno a la adecuada implementación de la tamización neonatal en Colombia mediante la aplicación de una encuesta en el marco de una investigación interpretativa basada en el método empírico-analítico ^20^.

## Materiales y métodos

Para la obtención de los datos, se recurrió a una encuesta diligenciada de forma anónima por los funcionarios involucrados en actividades de coordinación o vigilancia de programas de salud pública o de investigación en el Instituto Nacional de Salud que aceptaron participar voluntariamente. Se escogió a esta población dado que la tamización neonatal es un programa de salud pública de tipo asistencial con implicaciones en la investigación ^20^. Previamente se obtuvo la aprobación de los responsables de las direcciones de Vigilancia, de Redes y de Investigaciones del Instituto para abordar de forma directa a los funcionarios. Una vez diligenciadas, las encuestas se numeraron en orden ascendente. El cuestionario solicitaba señalar con una X la respuesta que se consideraba más acertada, por lo que no se consideraron para el análisis preguntas con más de una alternativa de respuesta, o ninguna.

El cuestionario traía 13 preguntas y un listado de posibles respuestas elaboradas con base en aspectos controversiales basados en principios bioéticos relacionados con la tamización neonatal. Las primeras 11 preguntas aludían a las pruebas para la detección de anomalías que pueden tratarse precozmente en beneficio del menor y, las dos últimas, a aquellas pruebas para detectar anomalías poco comprendidas o sin tratamiento que beneficie directamente al neonato. Además, se daba a los encuestados la posibilidad de explicar cada una de las respuestas mediante la libre manifestación de sus creencias y valores, si así lo deseaban. En el cuestionario también se solicitaba información relacionada con la experiencia del encuestado a lo largo de sus años en la profesión, en el cargo, y en los programas y actividades relacionadas con aspectos bioéticos o directamente con la tamización neonatal.

Una vez finalizada la etapa de recolección de encuestas, los datos de las respuestas se registraron en una hoja electrónica de cálculo. Todas las preguntas correspondían a variables pertenecientes a tres categorías: obligatoriedad, confidencialidad y anomalías poco comprendidas. En primer lugar, se obtuvo la información descriptiva, en tanto que los datos numéricos se expresaron en medias, medianas y rangos, y los categóricos, en porcentajes. Además, se hizo un análisis bivariado utilizando la prueba de ji al cuadrado cuando se relacionaban dos variables categóricas y, la prueba t de Student, para variables independientes en que una numérica se relacionaba con una categórica de tipo dicotómico. Para este análisis estadístico se utilizó el programa SPSS™.

En segundo lugar, se procedió a analizar las respuestas en texto libre mediante un enfoque interpretativo basado en la deliberación ^21^. En resumen, se utilizó un enfoque inductivo a partir de la lectura detallada para extraer del texto los valores expresados por los encuestados, entendidos como aquellas cualidades que corresponden a apreciaciones compartidas y reconocidas o con mérito por sí mismas (valores intrínsecos), o útiles para un fin (valores instrumentales). Posteriormente, se registraron en una hoja de cálculo asignándolas a cada una de las 13 preguntas del cuestionario, de tal manera que cada curso de acción quedara asociado a múltiples valores.

Por último, mediante un enfoque heurístico basado en la combinación de cursos de acción, se definieron como "deberes en un contexto colombiano" aquellas acciones que rescataban los valores expuestos por los encuestados escogiendo la mejor de cinco alternativas, como mínimo, en casos de controversia ^22^.

## Resultados

De un total de 127 funcionarios, 88 aceptaron participar y diligenciaron la encuesta de forma válida. La mayoría (64,2 %) de los encuestados pertenecía al sexo femenino, y 84,1 % de ellos ejercía profesiones en el campo de las ciencias de la vida. Se destacó una participación mayoritaria de profesionales de la salud humana, principalmente bacteriología, medicina y enfermería, profesiones que abarcaron el 54,5 % de la muestra. Los profesionales de áreas diferentes a la salud, como químicos, ingenieros, diseñadores e, incluso, un economista, agruparon al 13,6 % de la muestra. Los encuestados tenían, en promedio, 7,4 años de experiencia en el cargo y 4,8 años de experiencia en la implementación de programas. Aquellos con experiencia en labores específicamente relacionadas con aspectos éticos fueron pocos, y menos aún los que habían trabajado en tamización neonatal, aunque algunos de ellos tenían una amplia experiencia, de 16 a 20 años, en estos dos campos (cuadro 1).

**Cuadro 1:** Experiencia laboral de los funcionarios participantes involucrados en actividades de vigilancia, coordinación e investigación en el Instituto Nacional de Salud

Variable	Media	Mediana	Rango
Años en la profesión	13,3	12	(0-35)
Años en el cargo	7,4	4	(0-35)
Años en implementación de programas	4,8	2	(0-35)
Años en aspectos bioéticos	2,1	0	(0-20)
Años en tamización neonatal	0,57	0	(0-16)

En cuanto a la tamización con beneficio directo para el neonato, el análisis contempló dos categorías: en la primera, relacionada con la "obligatoriedad", más del 80 % de los funcionarios estuvo de acuerdo en efectuar la tamización independientemente del costo de oportunidad y mediante la oferta obligatoria del Estado (cuadro 2). Las respuestas libres de los funcionarios evidenciaron que, con esta postura, se rescataban valores intrínsecos de la salud, la calidad de vida y la equidad. Además, los encuestados también destacaron los valores instrumentales de la responsabilidad gubernamental, la amplia cobertura y la efectividad, señalando algunos que el costo es menor a largo plazo.

**Cuadro 2:** Opiniones con respecto a las controversias bioéticas en la categoría de obligatoriedad de la tamización neonatal con beneficio directo para el neonato

Variable	Sí	Porcentaje
Tamización independiente del costo de oportunidad	79	89,8
Consentimiento para uso de muestras residuales en investigación	72	81,8
Oferta obligatoria por parte del Estado (nación)	71	80,7
Consentimiento para uso de resultados en investigación	63	71,6
Consentimiento informado para autorizar tamización	56	63,6
Información detallada previa a la tamización	50	56,8

Asimismo, más del 80 % estuvo de acuerdo en la necesidad de solicitar el consentimiento para el uso de las muestras en la investigación, siendo un poco menor el porcentaje en torno a la necesidad de la autorización para el uso de los resultados en la investigación (cuadro 2). Se resalta que siempre que se consideró necesario el consentimiento para el uso de los resultados, también se lo consideró necesario para el uso de las muestras residuales en la investigación, pero no así lo contrario, es decir, se reconoció como mayor la necesidad de una autorización para el uso del material biológico. En cuanto a los valores, se conceptuó que el consentimiento informado para usar los resultados o las muestras residuales en la investigación entraña los valores de transparencia, autonomía y no maleficencia por evitar daños no justiciados al neonato. Por otro lado, los encuestados opinaron que el beneficio social de la investigación podría verse limitado por el requisito del consentimiento informado.

En la misma categoría, más del 35 % de los funcionarios estuvo en desacuerdo con el requisito de la autorización para hacer la tamización con beneficio directo para el neonato (cuadro 2). Se evidenció una tendencia a que los profesionales de campos diferentes a la salud y la biología consideraban más necesario el consentimiento (cuadro 3). Por el contrario, la opinión de que no es necesario el consentimiento fue cuatro veces más frecuente entre los profesionales relacionados con el cuidado directo del paciente, es decir, de enfermería, que entre el resto de los encuestados (OR=4,96; IC_95_% 1,3418,22; p=0,019). Además, tenían mayor experiencia en su profesión aquellos funcionarios que no consideraron necesario el consentimiento para la realización de la tamización neonatal (17,7 *Vs.* 11,79 años; p=0,007).

**Cuadro 3:** Distribución porcentual de las profesiones de los encuestados y su relación con el consentimiento informado

Profesión	Número	Porcentaje	Consentimiento informado^1^ %
Bacteriología	19	21,6	68,4
Medicina	17	19,3	64,7
Enfermería	12	13,6	33,3
Biología	6	6,8	50,0
Odontología	5	5,7	80,0
Medicina veterinaria	5	5,7	80,0
Nutrición	3	3,4	66,7
Otras profesiones de la salud^2^	4	4,5	75,0
No responden^3^	3	3,4	N.A.
Otras profesiones^4^	12	13,6	83,3
Administrativos	2	2,3	100,0
	88	100,0	

^1^ Porcentaje a favor del consentimiento informado en cada profesión

^2^ Terapia, instrumentación y enfermería auxiliar

^3^ Correspondientes a dos enfermeros y un biólogo

^4^ Profesiones diferentes a las del área de la salud y la biología: química, ingeniería, diseño, economía

Quienes no consideraron necesario dicho consentimiento argumentaron hacerlo a nombre de los valores de la salud, el bienestar y la calidad de vida de los afectados. Por el contrario, quienes lo consideraron necesario argumentaron el respeto a los valores de participación de los padres y su autonomía, además de la transparencia. Cabe mencionar que la opinión de que no es necesario el consentimiento informado para autorizar la tamización neonatal fue ocho veces más frecuente entre quienes creen que esta debe ser obligatoria y a cargo del Estado (OR=8,6; IC_95_% 1,07-70,02; p=0,028), lo cual evidencia la congruencia en las respuestas de quienes consideran que lo prioritario es actuar para evitar un daño.

También en la categoría de obligatoriedad se observó el desacuerdo con respecto al tipo de información que debe proporcionarse antes de la tamización neonatal. La mitad de los encuestados consideró que esta debe ser detallada y la otra mitad, que debe ser general (cuadro 2). A favor de la información general se argumentaron los valores de una fácil comprensión y su utilidad, dada la dificultad para entender en detalle el creciente número de anomalías. Por el contrario, se consideró que la exactitud de la información detallada era un valor para una adecuada adopción de decisiones.

En la segunda categoría, correspondiente a la confidencialidad de la tamización con beneficio directo para el neonato, más del 80 % de los encuestados consideró que todos los resultados, incluidos los de los portadores sanos, debían ser revelados a los padres, y casi el 80 % opinó que todos los resultados debían consignarse en la historia clínica (cuadro 4). Quienes propusieron entregar la totalidad de los resultados a los padres eran funcionarios con una mayor experiencia en tamización neonatal (8,6 *Vs.* 0,6 meses en promedio; p=0,019) y en el manejo de sus aspectos bioéticos (2,6 *Vs.* 1 año en promedio; p=0,023) que quienes proponían entregarlos solo parcialmente. En cuanto a los valores, se consideró que la entrega únicamente de los resultados anormales de utilidad para el manejo del tamizado respondía a los de eficacia de la detección y no maleficencia debido a que se mantenían ocultos otros resultados que podrían estigmatizar a pesar de no beneficiar al neonato tamizado. Por el contrario, la entrega total de los resultados rescata los valores de verdad y seguimiento cercano por parte de los padres y los médicos tratantes. Además, la consignación de todos los resultados en la historia clínica se funda en el valor instrumental de protección de la información y de un manejo manejo integral si se hace una adecuada interpretación.

**Cuadro 4:** Opiniones con respecto a controversias bioéticas en la categoría de conidencialidad en la tamización neonatal con beneicio directo para el neonato

Variable	Sí	Porcentaje
Revelación a los padres del estado de portador sano	84	95,5
Entrega de todos los resultados a los padres	73	83,0
Envío de todos los resultados a la historia clínica	68	77,3
Almacenamiento de muestras a mediano y largo plazo	61	69,3
Seguimiento sin ocultar la identificación del neonato	57	64,8

En cuanto al tipo de almacenamiento de las muestras residuales, casi el 70 % se mostró favorable a hacerlo a mediano y largo plazo, usualmente mayor a tres años ^9^, lo que los encuestados relacionaron con el valor del beneficio social por la generación de conocimiento. Por el contrario, aunque sin significación estadística, la mayoría de los miembros del Comité de Ética se mostró favorable al almacenamiento de las muestras residuales a corto plazo con base en los valores de privacidad, transparencia y eficiencia al disminuir la necesidad de recursos.

Hubo un menor acuerdo, de alrededor del 64 %, con respecto al seguimiento en salud pública sin ocultar la identificación del neonato tamizado (cuadro 4). En este sentido, se encontró que los funcionarios que proponían el uso de códigos para ocultar la identificación en el seguimiento con fines de salud pública tenían una mayor experiencia en aspectos bioéticos que aquellos que propusieron la identificación sin código (4,5 *Vs.* 1,26 años en promedio; p=0,009). El seguimiento con fines de salud pública sin esconder los datos de identificación fue relacionado por los funcionarios con el valor de la eficacia, en tanto que el uso de códigos lo fue con el de confidencialidad.

En cuanto a la tercera categoría, es decir, cuando la tamización no tiene un beneficio directo para el neonato, se observó que casi el 80 % de los encuestados estuvo de acuerdo en hacerla en el marco de investigaciones y no por sugerencia de eminencias (cuadro 5). Se consideró que ello no solo permite el beneficio social derivado del avance del conocimiento, sino que rescata los valores de eficiencia en el uso de los recursos, la autonomía mediante la participación de los padres adecuadamente informados, además de la beneficencia y la no maleficencia al equilibrar riesgo y beneficio.

**Cuadro 5:** Opiniones con respecto a controversias bioéticas relacionadas con la detección de anomalías poco comprendidas o sin tratamiento de beneficio comprobado para el neonato

Variable	Sí	Porcentaje
Tamización en el marco de una investigación	70	79,5
Revelación en todos los casos^1^	70	79,5

^1^ Independientemente de su utilidad para el manejo del neonato

Asimismo, casi el 80 % estuvo de acuerdo en revelar la totalidad de los resultados de la tamización, aunque no hubiera beneficio directo para el neonato y sin importar si su utilidad para el manejo médico hubiera sido demostrada (cuadro 5). Los encuestados consideraron que este proceder se cimentaba en los valores de transparencia y verdad. No obstante, una minoría consideró que solo deben revelarse los resultados cuando sean de beneficio para el neonato, o cuando este tenga edad para autorizarlo, lo cual se cimienta en el respeto a la intimidad.

### Deberes de la tamización en el contexto colombiano

En la categoría de obligatoriedad hubo acuerdo con respecto al deber estatal de la oferta obligatoria de la tamización neonatal con base en todos los valores propuestos por los encuestados, independientemente de la ignorancia, la falta de recursos de los padres o las diferencias culturales. En contraste, en esta misma categoría hubo controversia en cuanto a la necesidad del consentimiento informado para autorizar la tamización neonatal con beneficio directo para el neonato.

Asimismo, hubo controversia con respecto al tipo de información que debe proporcionarse antes de hacerla, por lo que se evaluaron las siguientes combinaciones de cursos de acción con base en los valores asociados:

1. Consentimiento informado e información detallada cimentados en los valores de autonomía, transparencia, participación y exactitud.
2. Tamización obligatoria sin consentimiento e información general en virtud de los valores de salud, bienestar, calidad de vida, fácil comprensión y utilidad en situaciones clínicas de falta de tiempo y múltiples focos de atención, como lo es el momento del nacimiento. La información general debe mencionar, no solo los beneficios, los riesgos y la necesidad de confirmación de un resultado positivo ^2^, sino también, la posible utilización de los resultados y las muestras de sangre en la investigación con lo que, incluso, se rescataría el valor de transparencia.
3. Tamización obligatoria sin consentimiento e información general con posibilidad de que sea detallada, esto en virtud no solo de los valores mencionados en el numeral 2, sino también, de la exactitud en respuesta a solicitudes específicas, aunque sin incluir la autonomía y la participación.
4. Consentimiento informado e información general con la posibilidad de que sea detallada ante una solicitud específica, con lo cual se rescatan los valores de autonomía, transparencia, participación, fácil comprensión, utilidad y exactitud, pero no los de salud, bienestar y calidad de vida.
5. Tamización obligatoria con posibilidad de rechazarla por razones específicas que tengan como in al neonato, e información general entregada antes de la tamización con posibilidad de detallarla ante un requisito específico, con lo que se rescatarían todos los valores planteados y se constituiría como un deber ser a partir de la deliberación en el contexto colombiano.

En cuanto al manejo de la información, no hubo acuerdo sobre la posibilidad de integrar la entrega de resultados y el seguimiento de casos positivos para alguna anomalía. En este sentido, se plantearon las siguientes combinaciones de cursos de acción:

1. Como punto de referencia se estableció la entrega parcial de los resultados anormales únicamente, lo que no permite un manejo integral ni un seguimiento cercano, valores instrumentales que facilitan la detección precoz de falsos negativos, como en algunos casos de fibrosis quística cuando no se identifica una segunda mutación ^23^, o déficits funcionales parciales como los descritos entre los portadores de la anemia de células falciformes ^24^. Además, su combinación con el seguimiento de casos sin ocultar la identificación pondría en riesgo la confidencialidad de la información.
2. El envío de todos los resultados a la historia clínica y su entrega completa a los padres, combinado con el seguimiento de casos sin ocultar la identificación, lo cual rescata, además de la eficacia de la detección, la protección de los datos en un documento sometido a reserva y el seguimiento cercano. Sin embargo, hay riesgo de no honrar la no maleficencia cuando se entregan incluso aquellos resultados cuyo equilibrio entre riesgo y beneficio es desfavorable para el neonato.
3. La entrega de todos los resultados y el seguimiento de los casos con identificación codiciada, lo que rescata el valor de la confidencialidad del seguimiento, pero requiere de un sistema ágil de acceso a la información por parte los responsables para satisfacer el principio de la eficacia de la intervención.
4. La consignación de todos los resultados en la historia clínica y su entrega a los padres acompañadas de un asesoramiento especializado y un seguimiento que garantice la codificación de la identidad, con lo que se rescata parcialmente la no maleficencia al reducir el riesgo de daños por falta de comprensión de los hallazgos
5. En este sentido, el envío de todos los resultados a la historia clínica y su posterior entrega completa a los padres mediante un asesoramiento especializado en el marco de un programa de salud pública que reconozca la importancia de la diversidad humana, así como un seguimiento que garantice la identificación codiciada y sea de rápido acceso para los responsables, se presentaría como el deber ser al cabo de la deliberación en el contexto real colombiano. Este curso de acción tiene la particularidad de reducir aún más el riesgo de daño sin justificación por el estigma o la discriminación en una sociedad en la que un alto porcentaje de individuos podría ser portador de mutaciones o tener variaciones correspondientes a hallazgos de significado incierto ^25^.

Por otra parte, hubo acuerdo en cuanto al deber de contar con el consentimiento informado para el uso en la investigación de las muestras residuales o los resultados de la tamización neonatal. En este contexto, el almacenamiento de las muestras con el consentimiento de los interesados por más de tres años no amenaza el respeto por la privacidad y la transparencia en su uso y honra el principio del beneficio social propio de la obtención de conocimiento en diferentes áreas. Sin embargo, debe reconocerse que se arriesga el valor de la eficiencia, pues implica costos adicionales que incluyen las locaciones y las condiciones requeridas, aunque en este sentido cabe mencionar que los programas de tamización neonatal en países desarrollados como el Reino Unido, donde se almacenan las muestras durante cinco años, por lo menos, han demostrado ser costo-efectivos ^23^.

En cuanto a las pruebas cuyos resultados no conlleven la posibilidad de una intervención temprana en beneficio del neonato, hubo acuerdo en que, para honrar los valores mencionados por los encuestados, estas deben realizarse en el marco de proyectos de investigación que garanticen el respeto por el recién nacido. Asimismo, al entregar los resultados en todos los casos, independientemente de que se haya demostrado el beneficio para el neonato mediante un estudio, o que este autorice su revelación al llegar a la edad necesaria, se rescatan los valores de transparencia y verdad. Sin embargo, los valores de autonomía e intimidad del neonato solo se respetarían si esta revelación se hace según un plan establecido en el marco de un estudio aprobado por un comité de ética de la investigación.

## Discusión

**Figura 1:**
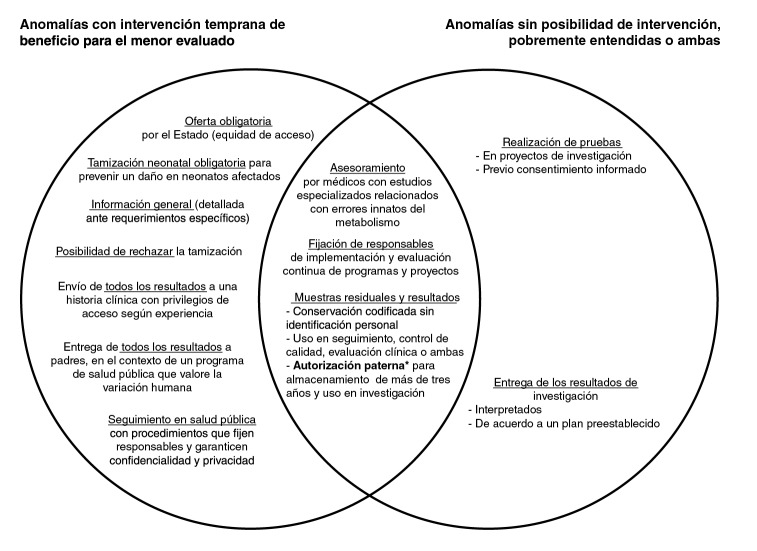
Propuesta de deberes en la práctica de la tamización neonatal en Colombia

En el ámbito local hubo acuerdo entre los funcionarios involucrados en la investigación y la vigilancia de programas de salud pública en torno a algunas de las controversias bioéticas sobre la forma de implementar la tamización neonatal. Sin embargo, el desacuerdo persistió en torno a otras de estas controversias, aunque fue posible encontrar cursos de acción basados en la deliberación que, en algunos casos, armonizaron incluso valores aparentemente opuestos. En consecuencia, fue posible hacer una propuesta para la implementación de la tamización neonatal en Colombia, complementando las características aceptadas con los deberes acordados por consenso (figura 1).

* Dispensable por un comité de ética independiente en investigación sin riesgo con resultados del tamizaje

No hubo controversia en cuanto a que la oferta de la tamización neonatal no debe depender del costo de oportunidad. Se consideró que el Estado debe garantizar el acceso de todo neonato a las pruebas para detectar anomalías cuya detección precoz permita un tratamiento de reconocida eficacia que disminuya la probabilidad de enfermedades que produzcan discapacidad o de muerte. En consecuencia, el criterio de justicia fue congruente con un Estado garante de derechos y concuerda con lo propuesto por la Organización Mundial de la Salud (OMS) ^26^ y lo promulgado en la Constitución Nacional y la normatividad colombiana sobre la tamización neonatal y los derechos de los niños ^17^^,^^27^^-^^29^. En este contexto, es fundamental evaluar cada prueba que se incluya en la tamización con base en el equilibrio entre riesgo y beneficio, el cual es desfavorable en anomalías leves o de inicio tardío en la vida, cuya detección no sería relevante en la atención neonatal pero podría provocar ansiedad en las familias, pues el recién nacido se convierte en un "paciente en espera" ^30^.

Por otra parte, la deliberación fue favorable a la tamización neonatal obligatoria con la posibilidad del disentimiento informado reconocido como válido y suficiente para rescatar la autonomía en cuanto a las pruebas y tratamientos que cumplan con los criterios de una tamización responsable ^24^, lo cual debe complementarse con otras consideraciones bioéticas como la de dar previamente una información general de fácil comprensión, pero con la posibilidad de ser detallada ante solicitudes específicas. En este contexto, es necesario un mecanismo normativo para asegurar que se dé la información en forma adecuada, considerando que la principal desventaja del disentimiento es que puede socavar los esfuerzos para garantizar que los padres estén adecuadamente enterados ^31^. Con este in, se pueden implementar diferentes estrategias ya probadas, como cursos de certificación obligatorios, auditorías, e información a la población ^32^, o requerir que el médico tratante deje constancia escrita de este hecho en la historia clínica, tal como se hace en el Reino Unido ^23^, y en concordancia con la vigente ley para la realización de la tamización neonatal ^15^.

En cuanto a la entrega de resultados de la tamización, se dio prioridad a la acción moralmente obligatoria de beneficencia al revelar todos los resultados para honrar el derecho a la vida y la salud de un individuo vulnerable comparada con la no maleficencia, que pretende evitar la discriminación comunicando solo los resultados anormales. Así, en un contexto de incertidumbre principalmente visibilizado por los funcionarios con mayor experiencia en tamización neonatal o en aspectos bioéticos, la deliberación evidenció que se confía en los padres como garantes del mejor interés para el menor ^33^ a pesar de su dificultad para mantener la confidencialidad en ambientes como el familiar, escolar o social ^34^. El ocultar información iría en contra del seguimiento cercano, cuyo fin es el neonato en sí mismo, es decir, en contra de su dignidad. Al respecto, es pertinente recordar que, en lo relacionado con la información, el derecho de referencia establece que no se pueden consentir prácticas contrarias a la dignidad ^35^.

Con este panorama, para el manejo adecuado de la información es fundamental un correcto asesoramiento que disminuya el riesgo de daños derivados del estigma o la discriminación. Debe contemplarse la posibilidad de restringir el acceso a los datos de la tamización neonatal; en este sentido, en algunos países se ha establecido el requisito de contar con profesionales especializados en genética médica para esta actividad ^6^. Por lo tanto, es recomendable adoptar medidas para preservar la confidencialidad de la historia clínica, blindándola con altos estándares de seguridad y procedimientos que estipulen los privilegios de cada usuario en atención a su carácter de documento privado y sometido a reserva ^36^. Por otra parte, la probabilidad de provocar daños debidos al estigma o la discriminación es aún menor si los programas de salud pública de crecimiento y desarrollo se articulan con la medicina personalizada que reconoce la diversidad humana como un valor. Un enfoque de salud pública es fundamental ante la posibilidad de medicalización de poblaciones sanas debido a la rotulación de gran parte de los individuos como genéticamente en riesgo ^34^.

En cuanto al seguimiento en salud pública, no se discute su importancia en los casos que han sido positivos en la tamización, pero hacerlo sin ocultar la identidad de los implicados pone en riesgo la confidencialidad de la información. En el Reino Unido, con excepción de la enfermedad de células falciformes y la talasemia ^23^, se reportan los resultados del diagnóstico de manera individual y anónima al organismo que coordina el programa de tamización, y en los laboratorios se conserva la información y se capturan los datos de seguimiento y sus resultados ^7^. En esta misma línea, los funcionarios con mayor tiempo de experiencia en aspectos éticos participantes en el presente estudio, recomendaron reflexionar en torno al uso de códigos en el seguimiento, de manera que quienes no tienen responsabilidades en el proceso, pero pueden tener acceso a los resultados en razón de sus funciones, no puedan identificar al individuo del cual provienen.

En cuanto al manejo de muestras residuales, es indiscutible el beneficio social derivado de resolver preocupaciones públicas, especialmente en el ámbito de agentes infecciosos, exposiciones ambientales, genética de poblaciones, exposiciones farmacológicas, defectos congénitos y discapacidad, así como en la introducción de nuevas pruebas ^31^. En el contexto colombiano de salud pública, se consideró necesario el consentimiento informado para el almacenamiento de las muestras y su utilización en la investigación, como sucede en otras poblaciones ^37^ y según lo estipulado por el derecho de referencia, aunque en dichas normas se otorgan facultades en el campo del derecho interno para reglamentar su utilización, especialmente por motivos de interés público ^38^. En este sentido, la alternativa éticamente aceptable es el disentimiento informado, el cual requiere que los padres hayan sido informados de manera comprensible y tengan la opción de rechazar la propuesta mediante un proceso que no sea dispendioso. Además, las muestras deben mantenerse anónimas, pero etiquetadas con códigos que permitan identificar al individuo del que provienen para comunicar hallazgos clínicamente valiosos cuando sea necesario ^31^.

Al igual que con las muestras residuales, la utilización de los resultados de la tamización neonatal con fines de investigación debe basarse en la obligación de no hacer daño injustificado, es decir, en el principio de no maleficencia ^21^, por lo que se justifica el requisito del consentimiento informado. Sin embargo, la dificultad para obtener la autorización de los padres lleva a plantearse cuán práctica puede ser esta recomendación habiendo la opción del disentimiento informado previo cumplimiento de los mismos requisitos establecidos para el uso de muestras, o de lo estipulado en la normatividad en cuanto a prescindir del consentimiento cuando se trata de estudios que utilizan los datos consignados en las historias clínicas pero no implican riesgo a juicio de un comité de ética de la investigación ^39^. La restricción para el uso de los resultados de la tamización neonatal sería menor que para el uso de muestras, ya que estas podrían permitir hallazgos que van más allá del ámbito de la tamización.

Por último, en el caso de la realización de pruebas sin beneficio directo para el neonato, la propuesta es admitirla en el marco de proyectos de investigación. Esto implica que el proyecto, incluido el consentimiento informado, debe ser aprobado por un comité de ética de investigaciones. Por otra parte, cuando la revelación de los resultados no repercute en el bienestar del neonato, es discutible el planteamiento de que los padres tienen el derecho a conocer la totalidad de los resultados en el marco del derecho fundamental a la intimidad por el cual típicamente opta la norma ^40^. En este contexto, un plan de entrega de la información revisado por un comité de ética de la investigación y debidamente consentido por los padres respetaría tanto su derecho a la información como el derecho a la intimidad del neonato.

Se pudo apreciar que el enfoque deliberativo permite proponer soluciones a controversias internacionales que han persistido, y cuyas implicaciones han justificado el pronunciamiento de la ley sobre lo que puede o no hacerse. En el contexto de la revelación de información, se cuestiona, no solo la entrega de todos los resultados, sino, incluso, el seguimiento tradicional que no oculta la identidad de los sujetos en aras de la eficacia. Se trata, en este caso, de la intimidad de la persona, su ADN e, incluso, de sus posibilidades de tener descendencia, por lo que está en juego el respeto de los derechos humanos ^41^. La necesidad del consentimiento informado se sustenta en el hecho de que los riesgos de la tamización neonatal no son triviales, pero se considera válido el principio legal de que incluso las intervenciones de alto riesgo pueden justificarse si se cuenta con autorización del sujeto. Otros daños que podrían derivarse de revelar información confidencial incluyen la disminución de las oportunidades de empleo, de aseguramiento y de procreación, así como la presencia de ansiedad prolongada ^24^. En otro sentido, hay detractores del uso del consentimiento que argumentan el alto riesgo que implica para la salud y la vida del neonato el rechazo de la tamización neonatal por parte de los padres cuando se trata de anomalías para las cuales hay tratamientos con un claro beneficio ^30^.

En síntesis, entre los profesionales relacionados de manera estratégica con la implementación local de la tamización neonatal, y con larga vinculación a la investigación o los programas de salud pública, no hubo controversia en cuanto a que el Estado debe garantizarla cuando hay claros beneficios para el neonato y que, más allá de los costos, es necesario evaluar el equilibro entre riesgos y beneficios independientemente de los intereses de los grupos de presión.

Por otra parte, a pesar del conflicto entre los principios éticos de no maleficencia y beneficencia, hubo acuerdo sobre la necesidad de revelar toda la información de la tamización neonatal mediante estrategias basadas en la deliberación, con el fin de rescatar los valores involucrados teniendo como referente la dignidad del recién nacido. También, hubo acuerdo en la necesidad del consentimiento para el uso de los resultados y las muestras residuales en la investigación, pero planteando alternativas de aplicación que concilien el respeto por los principios éticos y su utilidad práctica.

Po otro lado, a pesar de la falta de acuerdo sobre el tipo de información que debe revelarse y la necesidad de autorización de los padres para realizar la tamización neonatal de reconocido beneficio para el neonato, y dada la debida consideración al seguimiento en salud pública, se plantearon estrategias que rescatan la adecuada apropiación de la doctrina del consentimiento informado y la confidencialidad, todo ello en el marco de la reflexión sobre la implementación de la tamización neonatal en nuestro medio, respetando integralmente los aspectos éticos involucrados.
